# Cisplatin therapy and the problem of gender-related nephrotoxicity

**Published:** 2013-07-01

**Authors:** Hamid Nasri

**Affiliations:** Department of Nephrology, Division of Nephropathology, Isfahan University of Medical Sciences, Isfahan, Iran

**Keywords:** Cisplatin, Acute Kidney Injury, Renoprotection

**Implication for health policy/practice/research/medical education:**

It is well understood that there is a sex difference in the cisplatin-induced kidney toxicity in preclinical studies. However, few studies published to define sex difference on cisplatin nephrotoxicity. In this regard, to better understanding the factor of gender difference in cisplatin nephrotoxicity, more experimental rat model or clinical studies suggests.

It is established that cisplatin therapy is accompanied by damage to the kidneys (1). However, cisplatin is an applicable antineoplastic agent, against solid tumors in clinic (1,2). Cisplatin or cis-diamminedichloroplatinum II is an inorganic platinum-based antineoplastic agent (1-4). The renal toxicity of cisplatin therapy is considered to be one of its major complications (1-4). Studies showed that, 20-30% of the treated patients, experienced acute kidney injury while one third of cisplatin-treated patients end up with irreversible renal injury (1-5). Various mechanisms have been reported describing the pathogenesis of cisplatin-renal toxicity, consisting the production of inflammation, vascular injury, generation of free radicals, nephrotoxic metabolites and apoptotic pathways (3-6). In general, cisplatin damages the S3 segment of the proximal tubular cells, where it is preferentially accumulated (3-6). Current efforts to reduce kidney toxicity in patients receiving this drug, are still not satisfactory, as they can only partially prevent acute kidney injury (2-6). While a variety of substances either herbal or chemical drugs suggests having antioxidant efficacy and nephroprotective strategies are in develop, however recent studies revealed a gender related kidney toxicity for cisplatin (4-8).

Previously we noticed that, estrogen diminishes protective effect of erythropoietin against cisplatin-induced kidney toxicity in ovariectomized rats (9). In an another preclinical investigation to found the protective effects of endogenous nitric oxide donor on cisplatin-induced kidney toxicity, we noticed that L-arginine had protective property against cisplatin-induced renal damages in males, however it promotes the induced damages in females. We described a gender related difference in rat model of cisplatin-induced kidney toxicity (10). While, the role of sex in cisplatin nephrotoxicity is ill-understood, we recently conducted another investigation on rat model of cisplatin nephrotoxicity. We observed, losartan may prevent cisplatin nephrotoxicity in males, but it promotes the cisplatin-tubular injury in female rats. Likewise, we recently observed that, Vitamin E, Vitamin C, or losartan have not defending property against cisplatin nephrotoxicity in presence of estrogen in ovariectomized rat model (11), which is in agreement with our previous findings. More recently, we noticed that, erythropoietin as a kidney protective substance (1-6), may lead to different responses against cisplatin-kidney injury in male and female rat model. We found that, treatment by recombinant human erythropoietin (Eprex) significantly abolished changes in blood urea nitrogen and creatinine levels in male rats, conversely, the protective property of Eprex was not seen in females (12,13). In agreement to this investigation, we recently observed that, co-treatment of NO synthase inhibitor and cisplatin did not recover the increased the levels of serum creatinine and blood urea nitrogen in male rats but not in female rats (14). We also observed that cisplatin lonely increased kidney damage significantly, however, the injury induced by combination of cisplatin and NO synthase inhibitor was gender-related too (14). Thus we interpreted that NOS inhibition by NO synthase inhibitor increased cisplatin-induced renal injury, which was sex-related (14). Hence, it is well recognized that there is a gender difference in the cisplatin-induced renal injury in experimental investigations (13-15). However, scarce studies published regarding gender difference on cisplatin renal injury. In this regard, to better understanding the factor of gender difference in cisplatin kidney injury, more experimental rat model or clinical investigations suggests.

## Author’s Contribution

HN is the single author of the manuscript.

## Conflict of interests

None to declare.

## Ethical considerations

Ethical issues (including plagiarism, data fabrication, and duplicate publication) have been completely observed by the author.

## Funding/Support

No financial support by any institution.

## References

[1] Nasri H (2013). C-Phycocyanin attenuates cisplatin-induced nephrotoxicity in mice. Ren Fail.

[2] Nasri H (2013). World kidney day 2013: acute kidney injury; a public health aware. Iran J Public Health.

[3] Nasri H (2013). Reply: Oxytocin ameliorates cisplatin-induced nephrotoxicity in Wistar rats. Ann Saudi Med.

[4] Nasri H (2013). Protective effects of subchronic caffeine administration on cisplatin induced urogenital toxicity in male mice. Indian J Exp Biol.

[5] Nasri H (2013). Ellagic acid protects against cisplatin-induced nephrotoxicity in rats: a dose-dependent study. Eur Rev Med Pharmacol Sci.

[6] Nematbakhsh M, Nasri H (2013). Re: Effect of alcoholic extract of Nigella sativa on cisplatin-induced toxicity in rat. Iran J Kidney Dis.

[7] Rafieian-Kopaie M, Baradaran A (2013). Plants antioxidants: From laboratory to clinic. J Nephropathol.

[8] Nematbakhsh M, Ashrafi F, Nasri H, Talebi A, Pezeshki Z, Eshraghi F, Haghighi M (2013). A model for prediction of cisplatin induced nephrotoxicity by kidney weight in experimental rats. J Res Med Sci.

[9] Eshraghi-Jazi F, Nematbakhsh M, Pezeshki Z, Nasri H, Talebi A, Safari T (2013). Sex differences in protective effect of recombinant human erythropoietin against cisplatin-induced nephrotoxicity in rats. Iran J Kidney Dis.

[10] Eshraghi-Jazi F, Nematbakhsh M, Nasri H, Talebi A, Haghighi M, Pezeshki Z (2011). The protective role of endogenous nitric oxide donor (L-arginine) in cisplatin-induced nephrotoxicity: Gender related differences in rat model. J Res Med Sci.

[11] Nematbakhsh M, Ashrafi F, Safari T, Talebi A, Nasri H, Mortazavi M (2012). Administration of vitamin E and losartan as prophylaxes in cisplatin-induced nephrotoxicity model in rats. J Nephrol.

[12] Nematbakhsh M, Ebrahimian S, Tooyserkani M, Eshraghi-Jazi F, Talebi A, Ashrafi F (2013). Gender Difference in Cisplatin-Induced Nephrotoxicity in a Rat Model: Greater Intensity of Damage in Male Than Female. Nephro Urol Mon.

[13] Pezeshki Z, Nematbakhsh M, Mazaheri S, Eshraghi-Jazi F, Talebi A, Nasri H (2012). Estrogen Abolishes Protective Effect of Erythropoietin against Cisplatin-Induced Nephrotoxicity in Ovariectomized Rats. ISRN Oncol.

[14] Moslemi F, Nematbakhsh M, Eshraghi-Jazi F, Talebi A, Nasri H, Ashrafi F (2013). Inhibition of Nitric Oxide Synthase by L-NAME Promotes Cisplatin-Induced Nephrotoxicity in Male Rats. ISRN Toxicol.

[15] Pezeshki Z, Nematbakhsh M, Nasri H, Talebi A, Pilehvarian AA, Safari T (2013). Evidence against protective role of sex hormone estrogen in Cisplatin-induced nephrotoxicity in ovarectomized rat model. Toxicol Int.

